# Bridging the ACP literacy gap in Nigeria: A call for community and cross-sector collaboration

**DOI:** 10.1017/S1478951525100631

**Published:** 2025-09-12

**Authors:** Rikas Saputra, Isnaria Rizki Hayati, Neni Noviza, Yenni Lidyawati

**Affiliations:** 1 Department of Islamic Guidance and Counseling, Universitas Islam Negeri Raden Fatah Palembang, Palembang, South Sumatera, Indonesia; 2 Department of Guidance and Counseling, Universitas Riau, Palembang, Indonesia; 3 Department Indonesian Language and Literature Education, Universitas Sriwijaya, Palembang, South Sumatera, Indonesia

Dear Editor,

We read with great interest the article by Cadmus et al. (2024) titled “Knowledge and perception of older adults toward end of life and advanced directive in Nigeria,” published in *Palliative and Supportive Care*. This study offers insights into Nigerian older adults’ low awareness and engagement regarding advanced care planning (ACP) and advanced directives. We want to provide reflective comments and suggestions to strengthen the discussion further.

First, the study successfully highlights the significant knowledge gap among older adults in Nigeria regarding end-of-life care, ACP, and legal documents such as living wills and power of attorney. With only about one-fifth of respondents having heard these terms, it is clear that public education and ACP socialization remain highly limited in Nigeria. The study underscores the importance of advocacy and community-based education, particularly through the influential role of religious leaders, who serve as the primary information source for respondents (Saputra et al. 2024a). Leveraging community and faith-based approaches is crucial to improving acceptance and understanding of ACP, especially in deeply religious societies like Nigeria.

Second, the article aptly addresses the impact of culture, religion, and family structures on end-of-life decision-making preferences (Saputra et al. 2024a). Most Nigerian older adults still rely heavily on family for care decisions, emphasizing collective decision-making and family traditions (Ene et al. 2022). This phenomenon is common in many other developing countries, including Indonesia, indicating the need for cross-cultural approaches to develop ACP interventions (Pohan et al. 2024).

Nevertheless, there are areas where the discussion could be strengthened. First, the article should discuss the implications of low ACP literacy on the quality of palliative care and the risk of unwanted medical interventions at the end of life (Jones et al. 2021). Second, although most respondents prefer home-based care at the end of life, the article does not detail structural or policy barriers that might hinder the realization of these preferences, such as the availability of home care services and financial support (Saputra et al. 2024b). Further qualitative research may be needed to explore these issues in depth.

Furthermore, efforts to enhance ACP should involve multi-sectoral collaboration: healthcare institutions, religious leaders, media, and policymakers must all work together to broaden ACP literacy and access. Educational materials should be adapted to local languages, cultural norms, and spiritual preferences for effective community engagement (Haynes et al. 2023).

In conclusion, we appreciate this article’s valuable contribution to filling the ACP knowledge gap in low- and middle-income countries. The study also offers important lessons for other countries, including Indonesia, to improve ACP literacy and implementation based on local cultural and spiritual contexts. It is hoped that collective, cross-sectoral efforts and community-focused education will ultimately enhance the quality of end-of-life care for older adults worldwide.
